# Sequencing, Mapping, and Analysis of 27,455 Maize Full-Length cDNAs

**DOI:** 10.1371/journal.pgen.1000740

**Published:** 2009-11-20

**Authors:** Carol Soderlund, Anne Descour, Dave Kudrna, Matthew Bomhoff, Lomax Boyd, Jennifer Currie, Angelina Angelova, Kristi Collura, Marina Wissotski, Elizabeth Ashley, Darren Morrow, John Fernandes, Virginia Walbot, Yeisoo Yu

**Affiliations:** 1 BIO5 Institute, University of Arizona, Tucson, Arizona, United States of America; 2 Arizona Genomics Institute, Department of Plant Sciences, University of Arizona, Tucson, Arizona, United States of America; 3 Department of Biology, Stanford University, Stanford, California, United States of America; The Salk Institute for Biological Studies, United States of America

## Abstract

Full-length cDNA (FLcDNA) sequencing establishes the precise primary structure of individual gene transcripts. From two libraries representing 27 B73 tissues and abiotic stress treatments, 27,455 high-quality FLcDNAs were sequenced. The average transcript length was 1.44 kb including 218 bases and 321 bases of 5′ and 3′ UTR, respectively, with 8.6% of the FLcDNAs encoding predicted proteins of fewer than 100 amino acids. Approximately 94% of the FLcDNAs were stringently mapped to the maize genome. Although nearly two-thirds of this genome is composed of transposable elements (TEs), only 5.6% of the FLcDNAs contained TE sequences in coding or UTR regions. Approximately 7.2% of the FLcDNAs are putative transcription factors, suggesting that rare transcripts are well-enriched in our FLcDNA set. Protein similarity searching identified 1,737 maize transcripts not present in rice, sorghum, *Arabidopsis*, or poplar annotated genes. A strict FLcDNA assembly generated 24,467 non-redundant sequences, of which 88% have non-maize protein matches. The FLcDNAs were also assembled with 41,759 FLcDNAs in GenBank from other projects, where semi-strict parameters were used to identify 13,368 potentially unique non-redundant sequences from this project. The libraries, ESTs, and FLcDNA sequences produced from this project are publicly available. The annotated EST and FLcDNA assemblies are available through the maize FLcDNA web resource (www.maizecdna.org).

## Author Summary

To complement the completion of sequencing the maize B73 genome, we sequenced 27,455 full-length cDNAs (FLcDNA) from two maize B73 libraries representing the gene transcripts from most tissues and common abiotic stress conditions. The FLcDNAs are beneficial in determining the exon/intron structure of genes by aligning them to the sequenced genome; 94% of our FLcDNAs aligned to the maize genome. The 27,455 FLcDNAs were compared to gene sequences for rice, sorghum, *Arabidopsis*, and poplar; 22,874 were found in all four sets, and 1,737 were unique to maize. Two-thirds of the maize genome is composed of a type of repetitive sequence called “transposable elements”; only 5.6% of the FLcDNA sequence contained any segment homologous to these repeats. In addition to our set, there are three other sets of maize FLcDNAs for a total of 69,306 gene transcripts, where many of them are from different maize lines (i.e. FLcDNAs often have only slight differences reflecting divergence). We assembled these together using parameters that would allow most alleles and recently diverged gene transcripts to align together, resulting in 46,739 unique gene transcripts.

## Introduction

*Zea mays* L. is one of the world’s most important crop plants as well as a model organism for analysis of the impact of transposable elements on genome structure and gene expression [1] and the premier example of allelic diversity within an organism [2]. The success of approximately 100 years of maize genetic analysis is based on the functional diploidy of many loci, as the loss of one gene function is sufficient to generate a scoreable phenotype. Most duplicated genes have diversified in function, yet retain 80–90% sequence similarity with their paralogs [3]. There is a surprisingly high occurrence of local gene duplications in flowering plants, compared to animal genomes; for example, 12.6–16.6% of loci in *Arabidopsis thaliana* are estimated to be adjacent to a gene family member [4], and this estimate increases to one-third of the genes in maize [5]. A subset of these local duplications are either very recent or have been corrected by recombination; the frequency of nearly identical neighboring genes is estimated to exceed 1% in maize [6]. Furthermore, the entire genome was duplicated approximately 5–12 million years ago (MYA) [7].

There are currently over 2 million maize ESTs in GenBank [8]; the majority of these ESTs are drawn from 10 inbred lines. Comparison of these sequences illustrates the high single nucleotide polymorphism (SNP) and indel (insertion-deletion) polymorphism present in diverse maize lines [3]. Given the intrinsic error in EST sequence data, the polymorphism among maize lines, and the relatively recent duplication of the entire maize genome, assembly of ESTs into gene models presents practical problems. Setting the criteria for sequence similarity too high will artificially “split” alleles of the same locus into separate genes, while setting the criteria too low will combine duplicated loci into a single model. Therefore, high quality full-length cDNA (FLcDNA) sequences are recognized as the best source for transcription annotation. By comparing them to the genomic sequence, intron/exon and transcription start and stop sites are readily identified. Polymorphisms between nearly identical duplicated genes found in FLcDNAs can confirm or correct genomic sequence assemblies as well.

The B73 maize genome has been sequenced [9] using the clone-by-clone approach from the BAC-based physical map [10],[11]. Four maize FLcDNA datasets are available from GenBank to aid genome annotation: (i) Jia et al. [12] generated 2,073 FLcDNAs from different stages of endosperm development in inbred W22; (ii) Lai et al. [13] generated 3,384 FLcDNAs from osmotically stressed seedlings line L. Han 21; (iii) Alexandrov et al. [14] generated 36,565 FLcDNAs using different tissues and treatments from diverse hybrids, of which 10,084 were determined to be high quality unique clones; and (iv) we have generated 27,455 FLcDNA from maize B73, as described herein. These four datasets make up the total 69,306 maize FLcDNAs currently in GenBank with the keyword ‘FLI-CDNA’.

Here we report: (i) the construction of library C to complement the existing library B, each containing mixtures of multiple organs, abiotic treatments, and developmental stages from the maize B73 inbred, the same line employed for the genome sequencing project; (ii) the bidirectional sequencing of 364,385 ESTs from the two libraries; (iii) full-length sequencing of 27,455 clones by primer walking and software automation; (iv) the analysis of the 27k sequences; (v) the assembly and analysis of both the 27k FLcDNAs from this study and the full set of 69k FLcDNAs from GenBank, using three different parameters sets for a total of six assemblies; and (vi) a queryable website www.maizecdna.org of the EST and FLcDNA assemblies discussed in this paper. An additional 7,903 ‘almost finished’ FLcDNA sequences, which were not submitted to GenBank because of incomplete finishing, are also available for a total of 35,358 FLcDNAs for this project.

## Results

### Sequencing 366k and assembling 358k ZM_BF ESTs

As described in Materials and Methods, two normalized libraries were generated, where library B was a mix of 16 reproductive and vegetative tissues, and library C included six seedling stress treatments plus normal seedlings, field-grown leaves, and three stages of anthers. For this project, 364,383 ESTs from libraries B and C were Sanger sequenced and submitted to GenBank. Before assembly, these were trimmed for low complexity sequences caused by polymerase slippage [15], resulting in the removal of 25,406 ESTs. A previous dataset of 19,027 ESTs had been sequenced from library B, which was added to our EST set. The statistics of the three EST datasets are shown in Table 1, where there were a total of 140,682 mate-pairs and 76,642 un-mated ESTs, representing 217,324 clones.

**Table 1:** ZM_BF ESTs after removal of slippage and poly(A).

	EST Prefixes			Total
	ZM_BFa^a^	ZB_BFb	ZM_BFc	
From library	B	B	C	B and C
# ESTs	19,027	227,558	111,421	358,006
# Mate-pairs	7,862	89,674	43,146	140,682
% 5′ ESTs	55%	56%	58%	56%
% 3′ ESTs	44%	42%	41%	42%
Average length	673	671	688	677

a These ESTs were sequenced as a preliminary project and were submitted to GenBank in 2002.

A total of 358,006 ESTs from library B and C were used for assembly, prefixed with ZM_BFa (19k, library B), ZM_BFb (227k, library B) and ZM_BFc (111k, library C). The ZM_BF ESTs were assembled with PAVE (Program for Assembling and Viewing ESTs, [16]), which joins contigs with shared mate-pairs that do not overlap into a single contig joined by a string of ‘n’. This resulted in a total of 33,139 contigs and 5,910 singletons, where 16,891 contigs were joined by n’s based on mate-pairs. Given that the most recent estimate for the number of genes is fewer than 42,000 [9], this set of ESTs should represent the majority of the maize transcripts. These contigs were used to select clones for full-length sequencing.

In the spring of 2008, all maize ESTs ≥150 bp were downloaded from GenBank, which resulted in 797,619 ESTs including the ZM_BF ESTs. The GenBank ESTs were assembled with PAVE, resulting in 51,202 contigs and 36,780 singletons. The number of contigs and singletons is much higher compared to the ZM_BF assembly because the proportion of mate-pairs was much lower, the ESTs are from different cultivars, and the quality of some sets of ESTs may be low. There were 10,618 contigs containing only ESTs from the ZM_BF libraries, representing 10k potential discovery genes from the B and C libraries.

### Analysis of 27k maize FLcDNA

As described in Materials and Methods, a software system was built to select clones automatically, incorporate new reads, align them to the previous read, and select the next primer for walking. A total of 27,455 FLcDNAs were completed and submitted to GenBank (BT033159–BT043475, BT053765–BT056230, BT060453–BT064635, BT064666–BT066304, BT066325–BT070215, BT083470–BT088428). The sequences were evaluated for contamination and 136 were removed as 26 matched rRNAs, 16 were bacteria, 76 were fungi, and 18 were vertebrate.

The average insert size of the 27,319 FLcDNAs was 1,441.8±542.5 bp, which represented 39.4 Mb of genic sequence. The largest insert length was 4,651 bp and the smallest was 156 bp. The average G+C content was 53.8%, which is similar to that of rice exons [17], and about 8.9% of the clones showed G+C content ≥65%.

The computation of the longest open reading frames (ORF) resulted in 87.6% FLcDNAs with ORFs ≥100 amino-acid (aa) residues with 328 aa on average and 8.6% with ORFs between 50 and 99 aa residues. For the FLcDNAs with ORFs ≥100 aa, the average lengths of the 5′ and 3′ untranslated regions (UTRs) were 218 bp and 321 bp, respectively. The average G+C content was 56.8% for the coding region, 58.3% for the 5′ UTR and a significantly lower 43.7% for the 3′ UTR. The biased G+C content observed here was consistent with previously reported content in rice [18] and maize [12]. The highest G+C content of the coding regions was 84.8%.

### TE and SSR repeats

A total of 2,309 known Poaceae (grass family) transposable elements (TEs) was detected by RepeatMasker in 1,539 (5.6%) of the 27k FLcDNAs. Of the detected sequence from TEs, 46% were DNA TEs, 46% were LTR (long terminal repeat) retrotransposons, and 7% were non-LTR class. As shown in Table 2, the TE locations of 1,149 FLcDNAs with ORFs ≥100 revealed that 61% of TE insertions (1,018 occurrences) were found in either the 5′ or 3′ UTRs, where the 3′ UTR showed 4–5 times higher TE insertions than the 5′ UTRs. Of the remaining 39% TE insertions, 447 fragmented TEs were detected in coding regions or overlapped with coding sequences. DNA elements occurred 1.6 times more frequently than LTRs in coding regions, even though such elements are much less frequent in the genome [9]. These TE-related sequences are very likely fixed attributes of the B73 alleles that yielded the FLcDNAs and nearly all are short sequence motifs that are remnants of a TE (data not shown). There were 113 FLcDNAs completely covered by 215 TE insertions, which are likely to be TE-related protein coding genes.

**Table 2:** TE analysis of the 27k FLcDNAs.

	LTR	DNA TE	Non-LTR	Total
**TE sequences in 1539 cDNA**				
# of insertions	1068	1073	168	2309
Length (kb)	227.5	370.2	59.8	657.5
**TE location in 1149 cDNAs ≥100aa**				
5′ UTR	75	93	30	198 (12%)
5′ UTR-CDS	55	51	4	110 (6%)
CDS	134	217	25	376 (22%)
CDS-3′ UTR	92	66	18	176 (10%)
3′ UTR	375	394	51	820 (49%)
Total	731	821	128	1680

The frequency and location of simple sequence repeats (SSR) were analyzed in FLcDNA sequences using RepeatMasker, where a total of 2,132 SSRs (repeat length ≥20 bp) were detected in 1,930 FLcDNAs. As shown in Table 3, the most abundant SSR was tri-nucleotide (51.5%), followed by penta- (18.1%), di- (14.8%) and tetra- (11.4%) nucleotide repeats. As described by Fujimori et al. [19], rice and Arabidopsis FLcDNAs contained much higher tri-nucleotide repeats (67% and 59%, respectively) than human (23%) and mouse (18%) and our results confirmed the trend despite differences in SSR detection methods. A strong bias was observed in UTRs versus coding regions, as 68.5% of the tri-nucleotides repeats occurred in coding regions whereas 92–94% of di-, tetra- and penta-nucleotide repeats were found in the 5′ or 3′ UTRs. This suggests that tri-nucleotide SSRs are preferentially selected to prevent frame shifts in coding regions.

**Table 3:** SSR analysis of the 27k FLcDNAs.

SSR location	Di-NR^a^	Tri-NR	Tetra-NR	Penta-NR	Hexa-NR	Total	%
5′ UTR	177	256	124	225	32	814	38.2%
5′ UTR-CDS	1	4	2	3	0	10	0.5%
CDS	17	752	15	22	40	846	39.7%
CDS-3′ UTR	0	1	1	6	0	8	0.4%
3′ UTR	121	85	102	130	16	454	21.3%
Total	316	1098	244	386	88	2132	
%	14.8%	51.5%	11.4%	18.1%	4.1%		

a NR = nucleotide repeat.

### Homolog genes in other plant genomes

**Figure 1:**
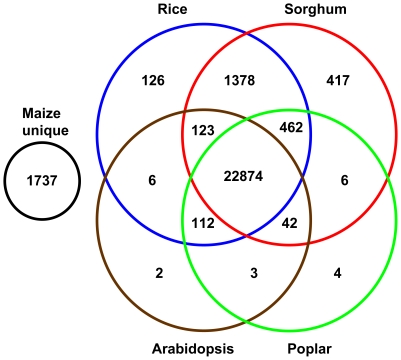
Homolog analysis with rice, sorghum, *Arabidopsis*, and poplar. The 27k FLcDNAs were searched against annotated protein gene models of each species, and the overlapping matches between species are displayed in the Venn diagram. Two overlaps (25 overlapping hits between rice and poplar, and two overlapping hits between *Arabidopsis* and sorghum) are not listed.

The 27k maize FLcDNA sequences were compared with annotated genes from the two monocot plants *Oryza sativa* ssp. japonica [17],[20] and *Sorghum bicolor* [21], and the two dicot plants *Arabidopsis thaliana* [22] and *Populus trichocarpa* [23]. Using BLASTx with e-value≤1e-10, maize homologs were found in the monocots sorghum and rice at 92.6% and 92.1%, respectively, and in the dicots Arabidopsis and poplar at 86.1% and 84.8%, respectively. A total of 25,582 FLcDNAs (93.6%) showed homology with at least one of the four gene sets, 22,874 (83.7%) were found in all four gene sets, and 1,737 (6.3%) did not have homology with any of the annotated genes (see Figure 1). Of the non-homologous FLcDNAs, 1,622 (93.4% of the 1,737) mapped to the maize sequenced genome suggesting that they may be unique maize genes, especially as sorghum and maize evolved as recently as 11.9 MYA [7].

Of the 1,622 putative unique maize genes, 147 FLcDNAs were removed from the list for having UniProt matches with e-value≤1e-10 from rice, sorghum, Arabidopsis or poplar proteins. The remaining 1,475 putative unique maize FLcDNAs were compared to 22,846 maize gene models associated with Gene Ontology (GO, [24]) terms, of which there were 90 unique GO terms. Of those, 779 FLcDNAs matched 820 maize gene models with GO terms. Interestingly about 64% of the 779 FLcDNAs encoded proteins with <100 amino acids.

Over- and under-representation analysis was performed using a hypergeometric distribution statistical test (p-value cutoff <0.05) in BiNGO [25] using the 820 GO terms associated with the putative unique FLcDNAs against all GO annotated maize genes. This analysis determined that 10 GO lineages comprised of 29 unique GO terms were significantly over-represented in 288 maize gene models, which were matched to 350 FLcDNAs (Table 4). The major over-represented GO descriptions from the biological processes were protein homo-oligomerization, response to freezing, and homoiothermy. The GO descriptions that were over-represented from molecular functions were water, ice binding, and ligand-dependent nuclear receptor activity. The over-representation of terms associated with cold tolerance may reflect the history of corn domestication. Maize was domesticated in Mexico nearly 10,000 years ago, and then moved by human activity into high temperate latitudes in both North and South America. In these new locations, cold spring and freezing autumn weather reduced the length of the growing season and likely resulted in rapid human selection for cold tolerance.

**Table 4:** Gene Ontology (GO) analysis of putative unique maize FLcDNAs.

Ontology	GO ID	GO lineage	FLcDNA count	Child GO description
Biological Process (GO:0008150)	GO:0044260	Cellular macromolecule metabolic process	3	lipoprotein metabolic process, lipoprotein biosynthetic process, protein amino acid lipidation, phosphoinositide biosynthetic process, GPI anchor biosynthetic process
	GO:0043933	Macromolecular complex subunit organization	23	macromolecular complex assembly, protein complex assembly, protein oligomerization, protein homooligomerization
	GO:0065007	Biological regulation	112	regulation of biological quality, homeostatic process, temperature homeostasis, homoiothermy
	GO:0032501	Multicellular organismal process	58	temperature homeostasis, homoiothermy
	GO:0051869	Response to stimulus	73	response to stress, response to abiotic stimulus, response to temperature stimulus, response to cold, response to freezing
Molecular Function (GO:0003674)	GO:0005488	Binding	53	water binding, ice binding
	GO:0030528	Transcription regulator activity	3	Transcription activator activity
	GO:0060089	Molecular transducer activity	20	Ligand-dependent nuclear receptor activity
	GO:0045735	Nutrient reservoir activity	4	Nutrient reservoir activity
	GO:0015457	Auxiliary transport protein activity	1	channel regulator activity, channel inhibitor activity, potassium channel regulator activity, ion channel inhibitor activity, potassium channel inhibitor activity

### Transcription factors

Putative transcription factor (TF) genes were identified by a BLASTx search against the rice and Arabidopsis transcription factor genes downloaded from PlantTFDB [26], identifying 1,965 (7.2%) putative TFs. Referring to predicted gene models, there are 2,383 TFs in rice and 1,922 TFs in Arabidopsis; if maize has a similar number of TFs, this indicates that our libraries are very well normalized as TFs are known to be rare transcripts and yet many are in the 27k set. Of the 1,965 TFs, the most abundant TF family was the bHLH group (7.2%) followed by the MYB (7%) and bZIP (6.5%) families. The top 10 maize TF families were similar to rice [27] and Arabidopsis [28] as listed in Table 5. However, the representation of the C2H2, AP2/EREBP and MADS families were slightly lower in maize (3.3%, 4.6%, 3.1%) compared to Arabidopsis (7%, 7.6%, 5.4%) [28]. In the less frequent TF families, FHA, LUG, SBP and GRF showed relatively higher frequency in maize (1.7∼2.1%) compared to Arabidopsis (0.1∼0.8%). There were no detected S1Fa-like, LFY, SAP, or NZZ families in the 27k FLcDNA set, which are also rare types in rice and Arabidopsis. Table S1 shows 60 TFs identified in the FLcDNAs.

**Table 5:** Top 10 putative transcription factors in the 27k FLcDNA set compared with rice and *Arabidopsis*.

	Maize		Japonica rice^a^		Arabidopsis^b^	
TF family	count	%^c^	count	%^d^	count	%^e^
bHLH	141	7.2%	184	7.7%	127	6.6%
MYB	137	7.0%	138	5.8%	150	7.8%
bZIP	127	6.5%	109	4.6%	72	3.7%
HB	103	5.2%	103	4.3%	87	4.5%
C3H	96	4.9%	90	3.8%	59	3.1%
AP2/EREBP	90	4.6%	182	7.6%	146	7.6%
NAC	85	4.3%	149	6.3%	107	5.6%
WRKY	82	4.2%	113	4.7%	72	3.7%
C2H2	64	3.3%	113	4.7%	134	7.0%
MADS	61	3.1%	83	3.5%	104	5.4%

a Gao et al. [27].

b Guo et al. [28].

c Percent of 1,965 putative TFs identified from the maize FLcDNAs.

d Percent of 2,383 TFs identified from the japonica rice annotated genes.

e Percent of 1,922 TFs identified from the Arabidopsis annotated genes.

### Protein function characterization

The non-redundant set was computed by assembling the 27k FLcDNAs with strict parameters (see Materials and Methods), resulting in 24,467 unique transcripts, which were searched with BLASTx against the UniProt plant database [29]. Ignoring maize matches (as most of them are from the maize FLcDNAs), 2,882 (11.8%) of the unique transcripts did not have a UniProt plant match using e-value≤1e-20; of the 21,585 (88.2%) with a match, 8,893 did not contain the word ‘putative’ in the description. Evaluating the set of 17,334 protein matches, 21% of them matched more than one unique transcript; these may be duplicated genes that have retained their function in both copies, yet diverged enough to not assemble together. Based on genetic analysis, many duplicated genes in maize have diverged in the time and place of transcription; thus, although each gene specifies the same function, mutation of either gene yields a unique phenotype [30],[31].

The 17,334 proteins that matched one or more transcripts mapped to 3,844 distinct GO terms, which mapped to 266 distinct GO Slim categories [32]. Table 6 shows the number of FLcDNAs mapped to the top 10 GO Slims, where each sequence can have more than one GO Slim term. The most abundant GO Slims were “metabolic” for the biological process, “membrane” for the cellular component, and “binding” for the molecular function, which were also found in high amounts in the maize FLcDNA set by Alexandrov et al. [14].

**Table 6:** Top 10 GO Slim annotations and number of FLcDNAs.

Biological		Cellular		Molecular	
metabolic process	4819	membrane	6359	binding	6150
cellular process	4785	nucleus	4162	catalytic activity	5326
response to stress	3873	plasma membrane	3849	protein binding	4933
biosynthetic process	2894	plastid	3395	transferase activity	4102
transport	2431	cytoplasm	2917	nucleotide binding	4076
transcription	2333	extracellular region	1886	hydrolase activity	3950
biological process	2059	intracellular	1862	DNA binding	2666
protein modification process	2034	mitochondrion	1450	oxidoreductase activity	2567
response to abiotic stimulus	1977	vacuole	1386	kinase activity	1973
catabolic process	1832	cell wall	1138	nucleic acid binding	1368

### Mapping the 27k FLcDNAs to the maize genome

**Figure 2:**
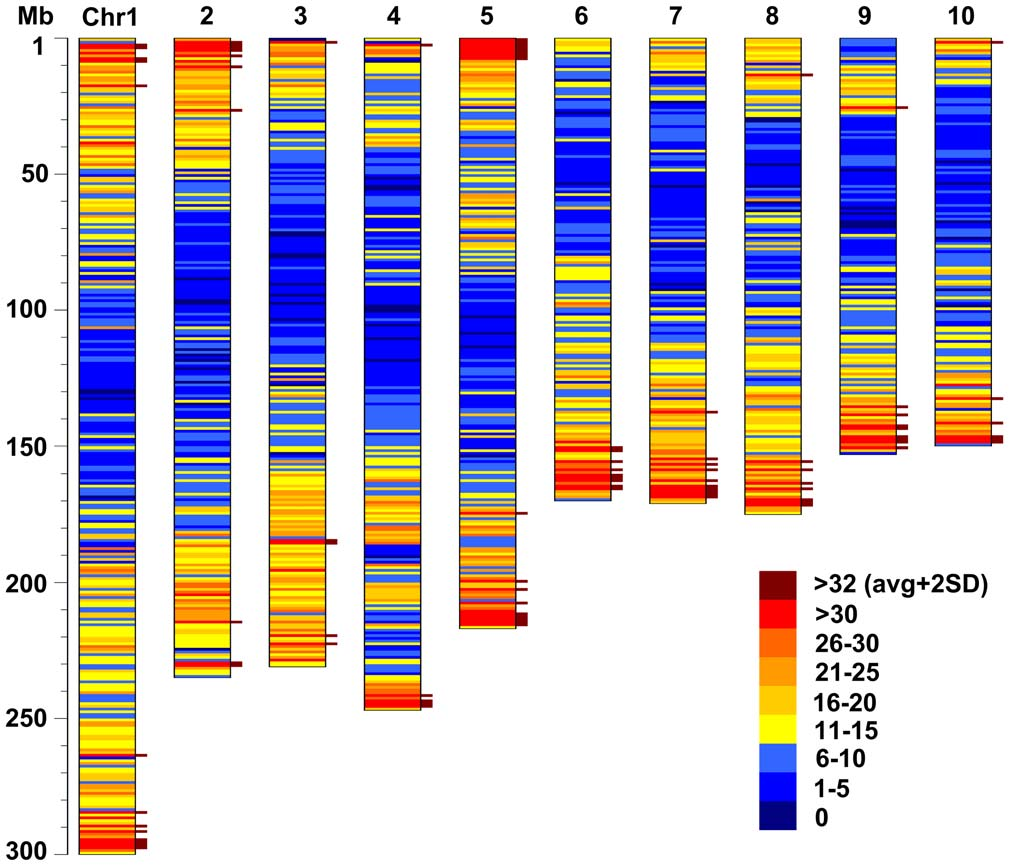
FLcDNA density heat-map displayed on the maize chromosomes. The 24,354 FLcDNAs that mapped to a single locus were counted in 1Mb bins (number of FLcDNA/Mb), color-coded, and plotted on the maize chromosomes. The yellow indicates average density (∼12cDNAs/Mb), the red is higher than average, and the blue is lower. The brown-colored bars next to each chromosome represent the regions where FLcDNA density is higher than average +2 standard deviations ( = 32 FLcDNAs/Mb).

A total of 25,753 FLcDNAs (∼94%) mapped to the maize sequenced chromosomes (B73 RefGen_v1, [9]) using BLAT [33]; results are summarized in Table 7. Retaining matches that had ≥97% identity and ≥80% alignment length (i.e. FLcDNA length coverage), 24,354 FLcDNAs mapped to a single locus and 1,399 (5.1%) mapped to two or more loci in the maize genome (excluding the 31 FLcDNAs that mapped to the unknown chromosome category). For the group that mapped to a single locus, the average density of the FLcDNA was 11.9 FLcDNAs/Mb or 84 kb/FLcDNA. Chromosome 5 showed the highest density at 13.5 FLcDNAs/Mb, and chromosome 4 was the lowest at 10 FLcDNAs/Mb (see Table S2). The single locus-mapped FLcDNAs tended to localize more in subtelomeric regions than the middle of the chromosome arms except for the short arm of chromosome 9 (see Figure 2). Anderson et al. [34] also observed the biased EST distribution on maize pachytene chromosomes using 1,195 genetically mapped EST markers and reported that 36% of ESTs were localized in the distal 20% of chromosome arms. The suppressed expression in the short arm of chromosome 9 could be associated with knob structure.

**Table 7:** Summary of FLcDNA mapping to the maize genome.

Total cDNAs	27,455	100.0%
Mapped	25,753	93.8%
Single locus	24,354	88.7%
Multi-loci	1,399	5.1%
Unmapped	1,531	5.6%
Homologs	1,417	5.2%
Unknown	114	0.4%
Mapped to unknown chromosome	35	0.1%
Contaminants	136	0.5%

For the 1,399 FLcDNAs that mapped to multiple loci in the genome, 67% mapped to two loci and 16% mapped to three loci. Ten FLcDNAs showed more than 100 mapping positions, of which eight had motifs that matched TE-related proteins (Gag and Pol proteins) and two had unknown protein function when compared to the UniProt database. Increasing the stringency to 99% identity and 95% alignment length, there were 531 FLcDNAs that mapped to two or more locations. Of this subset, 467 (1.7% of total FLcDNAs) mapped to exactly two loci in the genome indicating that they are very recently duplicated genes.

**Figure 3:**
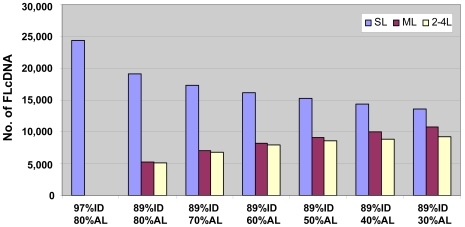
Detection of homeologous genes in the maize genome. Potential homeologous genes for the 24,354 single locus-mapped FLcDNAs were computed by using relaxed mapping parameters for aligning them to the maize genome. Approximately 44% SL-FLcDNAs had homeologous regions (ID; identity, AL; alignment length, SL; single locus, ML; multi loci (>4), 2–4L; 2–4 mapped loci).

The 24,354 FLcDNAs that mapped to a single locus (SL) were further examined for evidence of duplication by relaxing mapping parameters. With 89% identity and 80% alignment length, only 19% of SL-FLcDNAs aligned to homeologous locations (i.e. gene pairs that arose from polyploidization). With 30% alignment length, 44% of the SL-FLcDNAs detected homeologous regions of which 85% mapped to between two and four loci in the genome. Even with these extremely relaxed conditions, 56% failed to find homeologous regions (Figure 3). These results suggest that a higher proportion of duplicated genes are losing or have lost their functions by fragmentation, mutation and rearrangement mediated by illegitimate recombination [35] and helitron action [36] after the whole genome duplication in maize ∼11.9 MYA [7].

The analysis of the 1,531 unmapped FLcDNAs identified 1,417 plant homologs from the annotated genes in rice, sorghum, Arabidopsis and poplar genomes, which suggests that approximately 5.2% of genic regions are not represented by maize genome sequences. This result could reflect errors or missing sequence in the maize genome assembly, or alternatively, that the distinct sources of the B73 inbred line could harbor gene gain/loss events.

### Assembly of 27k and 69k maize FLcDNAs

The 69,306 maize FLcDNAs were downloaded from GenBank [8] and trimmed of poly(A) and slippage (see Materials and Methods), resulting in 69,214 sequences. In order to examine the relationship between all the available maize FLcDNAs, three stringencies of assemblies were performed with both the 27k FLcDNA set and the 69k trimmed FLcDNA set. The strict assembly required 100% identity with up to 10 ignored discrepant bases on either end (see Materials and Methods); the aim was to identify clones with practically identical sequence although such clones may vary on the start and end coordinates. The semi-strict assembly required 90% identity with up to 15 ignored end bases; the aim was to identify alleles and very recently diverged paralogs. The loose assembly allowed mixed orientation, 80% identity and up to 350 ignored end bases; the aim was to identify distant paralogs and anti-sense transcripts. Though the resulting contigs can contain different relations depending on the assembly (e.g. allele, paralog), we will use the term ‘unitrans’ (unique transcript) for the combined set of contigs and singletons.

Table 8 shows the results of assembling the 69k with the three sets of parameters, where the clones represent four FLcDNA projects performed with different lines. For the semi-strict assembly, where alleles and recently diverged transcripts should assemble together, there were 46,739 unitrans (33,226 singletons and 13,513 contigs). The table lists the number of contigs containing clones from each combination of the four projects (e.g. 104 contigs in the 69k semi-strict assembly have at least one FLcDNA from each project). For the semi-strict assembly, there were 13,368 unitrans with sequences only from the Yu-BC set, composed of 12,292 singletons and 1,076 contigs; 9,865 contigs had at least one sequence from both the Yu-BC set and another project; and 23,506 unitrans did not have a sequence from the Yu-BC set.

**Table 8:** Assemblies of four maize FLcDNA projects.

Project	#FLcDNA^a^	Line	Libraries
Yu-BC	27455	B73	Different tissues and treatments
Wang [12]	2073	Inbred W22	Endosperm development
Messing [13]	3370	Han 21	Osmotically stressed seedling
Feldman [14]	36316	Hybrid	Different tissues

	69k assemblies		
By projects^b^	Strict^c^	Semi^d^	Loose^e^
*Singletons*			
Y	21578	12292	6110
W	1761	889	494
M	3225	1271	423
F	32197	18774	8068
Total	58761	33226	15095
*Contigs*			
Y	1455	1076	1004
W	12	26	58
M	5	37	19
F	773	1667	2317
YW	94	89	47
YM	50	344	273
YF	1849	7937	9667
WM	2	5	1
WF	107	373	333
MF	51	438	445
YWM	0	0	0
YWF	68	480	697
YMF	24	900	1499
WMF	2	26	46
YWMF	1	104	212
Total	4495	13513	16620

a The 69,306 downloaded FLcDNA sequences were further trimmed with our scripts, which removed some clones from the assembled set.

b The first letter of each project name is used to indicate if there was at least one FlcDNA from the project in the contig.

c Strict assembly 1: 100% identity, ≤10 ignored end bases (see Materials and Methods).

d Semi-strict assembly 2: 90% identity, ≤15 ignored end bases.

e Loose assembly 3: Reverse orientation, 80% identity, ≤350 ignored end bases.

The lengths of the FLcDNAs and the assembled contigs are shown in Table 9, where in the 69k FLcDNAs, 232 of the 284 sequences greater than 3,000 bp are from the Yu_BC set. The 69k set has 171 FLcDNAs less than 100 bp, whereas the 27k set has none. Many of the smaller clones assembled in with larger clones as the stringency of assembly was reduced, which resulted in alternative start and end sites (as discussed below). Only the loose assemblies allowed reversed clones, where the 27k assembly had 146 reversed clones and the 69k assembly had 1,043. Table 10 lists the number of FLcDNAs per contig, where in the strict assemblies, 20% of the 27k FLcDNAs were in contigs and 15% of the 69k FLcDNAs were in contigs.

**Table 9:** Length of individual FLcDNA clones and assembled FLcDNA contigs.

		27k FLcDNA Assemblies				69k FLcDNA Assemblies		
Lengths in bases	27k FL cDNAs	Strict	Semi	Loose	69k FL cDNAs	Strict	Semi	Loose
<100	0	0	0	0	171	156	67	64
101–500	1052	685	614	435	5535	4992	2774	1046
501–1000	4552	3833	3644	2353	23530	21311	14676	7603
1001–1500	9903	9023	8536	6613	20317	18619	14007	9883
1501–2000	8251	7442	7071	6079	13680	12526	10077	8278
2001–2500	2768	2579	2470	2317	4585	4298	3822	3505
2501–3000	697	672	659	626	1112	1075	1027	1007
3001–3500	188	187	182	185	238	233	238	272
3501–4000	34	35	33	35	36	36	38	41
>4001	10	10	12	13	10	10	13	16
Total	27455	24467	23221	18656	69214	63256	46739	31715

**Table 10:** Analysis of assembled contigs.

	27k FLcDNA assemblies			69k FLcDNA assemblies		
	Strict	Semi	Loose	Strict	Semi	Loose
FLcDNA in contigs	5282	7373	14450	10453	35989	54119
Reversed^a^	0	0	146	0	0	1043
Total Contigs	2294	3139	5651	4495	13513	16620
2 clones	1823	2402	3669	3521	8422	7138
3–5 clones	451	700	1885	933	4681	8126
6–10 clones	20	37	95	38	382	1253
11–20 clones	0	0	1	3	26	95
21–50 clones	0	0	1	0	2	8
Contigs ≥4 FLcDNA^b^	150	230	731	311	2053	5341
SNPs	0	35	23263	0	4786	176626^c^
Contigs with SNPs	0	10	315	0	780	3317
GPs	0	13	360	0	935	41060^c^
Contigs with GPs	0	10	139	0	505	10889
with SNPs+GPs	0	4	111	0	423	1747
Alternative 5′ sites^d^	48%	49%	54%	44%	53%	60%
Clustered sites^e^	44%	26%	50%	39%	46%	52%
≥100 (# ≥2 FLc)^f^	31%(13)	31%(17)	32%(33)	22%(45)	26%(148)	29%(248)
≥50<100	7% (1)	7% (6)	9% (7)	8% (3)	7% (28)	9% (68)
≥25<50	5% (3)	5% (5)	7% (9)	8% (6)	8% (45)	9%(110)
≥10<25	5% (3)	5% (7)	6%(14)	9%(10)	10% (80)	10%(210)
Alternative 3′ sites^d^	44%	45%	52%	47%	54%	61%
Clustered sites^e^	33%	34%	40%	33%	37%	43%
≥100 (# ≥2 FLc)^f^	7% (1)	8% (1)	12% (8)	8% (5)	9% (37)	14%(115)
≥50<100	9% (6)	8%(10)	9%(18)	8%(13)	8% (55)	9%(138)
≥25<50	8% (4)	8% (9)	9%(18)	7%(10)	8% (69)	9%(199)
≥10<25	8% (9)	9%(17)	9%(38)	8%(22)	10%(168)	11%(413)

a Reverse complemented clones (only allowed in the loose assembly).

b SNPs and GPs (gap polymorphisms) were only identified in these contigs.

c Many of these SNPs and GPs for the loose assembly are in the end regions.

d Percentage of ends that are not the first/last two bases of the consensus sequence.

e Percentage of clustered ends, where each cluster contains the ends within 10 bases of another.

f Percentage of clusters ≥100 bases from the previous cluster (number of these that have at least 2 FLcDNAs in both clusters). The next three rows are the same but with different distances.

### SNPs, GPs, and alternative start/end sites

**Figure 4:**
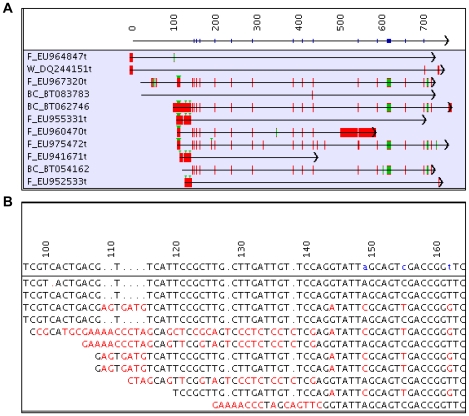
A contig in the 69k loose assembly with multiple SNPs and GPs. (A) The red vertical lines indicate a mismatch with the consensus sequence, and green vertical lines indicate a gap in relation to the consensus sequence. The clone prefix indicates the project (BC – Yu, W – Wang, M – Messing, F – Feldman). (B) Base view of the 5′ ends of 7 FLcDNAs with alternative start sites in relation to the consensus sequence. This is a close-up of bases 97–164 from the alignment shown in (A). The red bases do not agree with the consensus.

The 27k and 69k assemblies were analyzed for maize polymorphisms. As shown in Table 10, the 27k semi-strict assembly only had 35 SNPs (single nucleotide polymorphisms) detected in 10 contigs, whereas the 69k semi-strict assembly had 4,786 SNPs in 780 contigs. Furthermore, the 27k assembly had 13 GPs (gap polymorphisms) in 10 contigs and the 69k assembly had 935 GPs in 505 contigs. The high number of SNPs and GPs in the 69k assembly was expected, because the full set of FLcDNAs are from different maize strains. The 69k loose assembly had 176,626 SNPs and 41,060 GPs, where many of the polymorphisms were near the ends of the FLcDNAs; for example, Figure 4 shows a series of polymorphisms at the beginning of the clones.

The assemblies have a large number of small gaps between the clones aligned in the contigs. Table 11 shows the number of gaps of size 1 to 25. Although the number of gaps of a given size decreases overall with the size of the gap, there are peaks that tend to occur at multiples of 3. Within coding regions, these gaps will be reflected in gain/loss polymorphisms in the amino acid alignment of proteins. Figure 4 shows an example from the 69k loose assembly, where contig 00099 has a gap of size 9 at position 613 that is contained in four of the 11 FLcDNAs. Between bases 108 and 140 of the consensus sequence, seven FLcDNAs have start sites and there are 11 SNPs and 4 GPs (sizes 2, 4, 1, 1). Table S3 shows the distribution of the number of gaps per contig, where the 27k semi-strict assembly has 493 contigs with one gap, 82 with two gaps, and a quick decline in contigs with more gaps.

**Table 11:** Size and number of small gaps^a^.

	27k Assembly		69k assembly	
Gap Sizes	Semi	Loose	Semi	Loose
1	437	3294	9446	18439
2	156	1411	2470	5902
3	141	**1789**	2288	**6083**
4	58	417	919	2049
5	54	290	572	1236
6	**83**	**485**	**1073**	**2339**
7	32	154	359	733
8	21	100	**412**	802
9	**53**	**191**	373	**811**
10	21	53	121	279
11	14	37	95	203
12	**30**	**100**	**235**	**501**
13	14	27	71	155
14	**29**	39	70	128
15	21	**56**	**83**	**194**
16	20	23	53	95
17	10	21	17	49
18	5	**23**	**35**	**112**
19	5	8	6	29
20	3	18	4	52
21	1	17	5	72
22	1	6	5	40
23	0	6	2	27
24	0	8	2	52
25	1	6	4	28

a Bold text highlight counts that are greater than the previous.

The 27k and 69k assemblies show that many FLcDNAs have alternative start and/or end sites in relation to the other clones in the contigs. Several factors underlie this observation. First, some of the alternative sites could reflect incomplete clones. Second, some could occur if sequencing did not reach the end of the clone, especially on the 3′ end due to slippage. Third, the alternative sites could be alternative transcription initiation and polyadenylation sites. For example, Marrs and Walbot [37] showed that the *Bronze2* gene has two transcription initiation sites 200 bp apart. Additionally, Nash et al. [38] showed that the *Bronze2* gene had three distinct polyadenylation sites, which has also been found in an Arabidopsis homolog [39].

Figure 4 provides an interesting example of alternative start sites. There are two sets of clones, where the first set is the five clones without the large gap (at position 613) and the second set is the four clones with the gap (there are two clones that do not cover the gap). The first set has a strong alignment to position 714.9 Mb on chromosome 8 (B73 RefGen_v1, [9]), and the second set has a strong alignment downstream at 716.5 Mb. There is a potential alternative transcription initiation site for the first set, as two clones start at the beginning of the consensus sequence and two start over 100 bases downstream. There are no clones in the second set that start at the beginning of the consensus, and there are three clones with start sites over 100 bases downstream. Interestingly, there is a clone from each set that starts approximately 27 bases downstream from the beginning of the consensus and they have the exact same end position.

To give an indication of the diversity of the location of these sites for both the 27k and the 69k assemblies, the following percentages and numbers were computed (see Table 10): (i) the percentage of alternative ends that were not in the first/last two positions; (ii) the percentage of alternative clustered ends sites, where sites within 10 bases were clustered together; (iii) percentage of the clustered ends where the cluster was greater than a given distance (i.e. 100, 50, 25 and 10) from the previous cluster; and (iv) the number of possibly significant clustered ends where each cluster had at least two clones. In the two strict assemblies, any of these numbers could represent the possible number of alternative transcription initiation and polyadenylation sites, where the clusters containing at least two clones and start sites ≥50 bases apart are the most likely to be true alternative transcription initiation sites.

### The maize FLcDNA website

Many of the results in this paper can be further investigated from the Maize FLcDNA website (see the Analysis section in Materials and Methods). The PAVE software system has a queryable web interface, which has been used for query and display of the ZM_BF and GenBank EST assemblies, the strict assembly of the 27k set, all three assemblies for the 69k set, and a semi-strict assembly of the 69k clones along with the 7,903 unfinished clones. The GenBank EST contigs and 76k FLcDNAs have been aligned to the sequenced maize BACs, and are available from a genome browser. Additionally, the 76k maize FLcDNAs were aligned to the maize sequenced genome with cutoffs ≥1,000 base alignment and ≥95% identity, which resulted in 41,911 predicted genes. Using these FLcDNA-based gene models and the sequenced genome, version 3 of the SyMAP software [40] was used to compute synteny blocks between maize, sorghum [21] and rice [17],[20], where the alignments of the FLcDNAs to the sorghum and rice sequences may be viewed through the SyMAP java interface.

## Discussion

This initiative resulted in multiple important resources for the maize community to identify new genes, determine exon/intron structure of genes, and to characterize the transcriptome of maize. First, we produced two normalized libraries of maize B73 FLcDNAs from most tissues and common abiotic stress conditions; both the libraries and individual clones in a Gateway-compatible vector are available from the Arizona Genomics Institute (www.genome.arizona.edu/orders). Second, 364,383 bidirectional ESTs were submitted to GenBank. Third, 27,455 full-length cDNAs were submitted to GenBank. Fourth, three annotated assemblies of different stringencies of all 69k maize FlcDNAs are available from our website at www.maizecdna.org, along with 7,093 unfinished FLcDNAs. These resources were used in the annotation of the maize genome sequence [9], and will aid the assembly of unique transcripts in PlantGDB [41] and comparative analysis in Gramene [42].

## Materials and Methods

### Library construction

Seeds of the B73 inbred line were obtained from Pioneer Hi-Bred, Inc. (UT703 23 C30L) and propagated by self-pollination. The first library in this study was produced as part of a prior project where members of the Maize Gene Discovery Project collected 16 tissue types: seedling root, seedling shoot, inner husk, silks, 2 cm ear, 2 mm ear, adventitious root (stem tissue), 5 cm tassel branch with 0.5 mm anthers, 10–15 days after pollination (dap) embryos, 21 dap embryos, 21 dap aleurone, 7 dap whole kernel (primarily endosperm), adult leaf blade, adult leaf sheath, somatic shoot apex with four leaf primordia, and pollen. A full-length, normalized cDNA library, referred to as B, was constructed using these tissues (see details below). A second library in this study was produced using the same B73 source to fill in life stages and abiotic treatments missing in the first library. One suite of samples consisted of 7-day seedlings (with the endosperm removed) as normal control tissues plus 7-day seedlings subjected to heat, cold, salt, desiccation, UV-B, and lack of light [43]. The second suite of samples consisted of multiple stages of dissected anthers from the 1.0 mm length stage of rapid mitotic proliferation through the end of meiosis, approximately 10 days later. The final samples were intact juvenile leaf (blade, sheath, ligule-auricle region) and imbibed embryo and scutellum. A full-length, normalized cDNA library, referred to as C, was constructed using these tissues. The two normalized FLcDNA libraries are available as clones, whole libraries, or high-density hybridization filters from the AGI Resource Center (www.genome.arizona.edu/orders), where the B library is named ZM_BFa and ZM_BFb, and the C library is named ZM_BFc.

The libraries were constructed at Invitrogen (Carlsbad, USA) as follows: mRNA was isolated independently from the various tissues described above and 5′ capped-single strands were captured via first strand cDNA synthesis using 3′ oligo dT containing Gateway adapter attB2. Incomplete complimentary double strands, where the 5′ mRNA was retained without cDNA, were removed by RNase digestion followed by CAP binding protein selection and enrichment. After synthesis of second strand cDNA and ligation of attB1 the double-stranded cDNA were cloned. Each library was normalized using subtractive methodology where the abundant transcripts were biotinylated and used as drivers against circularized single strands of the same library. Following hybridization and purification using a streptavidin column and phenol cleanup, resultant DNAs were made double stranded and cloned into the pCMVSport6.1 vector before transformation into DH10B T1 T5 resistant *E. coli* (Pei-zhong Tang, Invitrogen Inc., personal communication; www.invitrogen.com).

### EST sequencing and assembly

FLcDNA clones for maize libraries B and C was picked with a Q-Bot (Genetics, UK) and arrayed into 384 well plates. Clones were inoculated in 200 µl of 2XYT media containing 50 µg/mL ampicillin and cultured at 37° C with 250 rpm overnight. Plasmid preparations were performed in a 96-deep-well block using a modified alkaline lysis method. The ESTs were sequenced using M13f and M13r primers and Big Dye Terminator (V3.1) sequencing chemistry (Applied Biosystems, USA). Post reaction clean-up used the Clean-Seq (Agencourt, USA) protocol with a BiomekFX (Beckman Coulter, USA) and the reactions were loaded on an ABI 3730XL (Applied Biosystems, USA). Primer walk sequencing was performed with the same protocol mentioned above with normalized custom primers in 384-well plate format (Invitrogen, USA).

The ESTs and primer walk sequences were base-called using Phred [44], and vector and low-quality bases were trimmed with Lucy [45]. The 364k ESTs submitted to GenBank were further trimmed using a customized bd2006trimmer [15] to remove the detectable slippage and any remaining poly(A) tail retained after Lucy trimming.

The ESTs were assembled with PAVE (Program for Assembling and Viewing ESTs, [16]). PAVE uses MegaBLAST [46] for clustering ESTs and performs iterative assemblies with CAP3 [47]. The iterative assembly allows mate-pairs to be retained in the same contig even if there were no overlapping mate-pairs in the contig. It also allowed the removal of redundant “buried” ESTs during assembly that were contained within another “parent” EST to avoid computational time and space problems. The buried clones were inserted into the parent’s contig after assembly, and are only shown in the web display of the contig alignment when requested by the user.

### Clone selection and walking for FLcDNA sequencing

Clone selection was performed using the PAVE assembly of the B and C libraries. An effort was made to select all potentially unique transcripts contained in the libraries. In addition, potential alternate start sites, alternate ends, and anti-sense transcripts were selected as full-length sequencing candidates.

A web-based software system was developed to track the history of every plate and clone. The system automatically performed the following seven tasks: (i) clones were selected for sequencing, as described below; (ii) selected clones were assigned to re-array plates sorted by original plate, as this minimized the number of times the original plates were pulled from the freezer; (iii) the 5′ and 3′ sequences were exported from a set of plates in order to be processed by the primer picking script. The resulting primers were entered into the system and the primers ordered from Invitrogen (Carlsbad, USA); (iv) the clones were sequenced off the primers and the resulting walked sequences entered into the system; (v) using dynamic programming, the walked sequences were aligned to the parent sequence. If the new sequence aligned, then the walked sequence would be used for priming the next walk; otherwise, the parent sequence was re-used but a different primer point was selected; (vi) steps iv and v were repeated until the clone passed the completion rules, as described below; and (vii) clones that did not pass the completion rules were entered into the finishing queue for manual review.

Clone selection for the initial plates required a high quality 5′ EST with a corresponding 3′ EST that appeared to have a poly(A) tail. The first plates were automatically walked from 5′ to 3′ and 3′ to 5′ simultaneously. Because of the poly(A) slippage problem, most plates of clones were selected based on high quality 5′ ESTs only, and the system was modified to walk first from 5′ to 3′, detect the 3′ vector (if possible) then reverse direction and walk back the 3′ to the 5′. Automatic walking was still difficult, because the software could often not identify the 3′ vector (to verify the 3′ end) because of slippage over the poly(A) tail. The end detection software was later modified to accept an apparent poly(A) tail with subsequent slippage as the turning point.

Primer design was performed in batch, calling the primer3 program [48] with parameters optimized for maize. Primer design codes were automatically assigned to sequences to indicate the desired priming location or to request a replacement for failed primers. Repetitive sequences were excluded. Sequences with no primers under the most stringent parameters were automatically queued for up to three progressively less stringent rounds of primer design; in only a few cases was manual primer design required.

### Completion rules and manual review

The software system initially required clones to have a 5′ and a 3′ supporting base for each position with a combined Phred value of ≥40 to be accepted as a complete FLcDNA. Clones were entered into the manual finishing queue if there were >10 base-pairs failing to meet the 40 combined Phred quality, or they had high quality discrepant bases after bi-directional walking, or if bi-directional walking could not be completed because of a poor quality 3′ EST. For manual finishing, all sequences (i.e. 5′ EST, 3′ EST, and walks) were re-assembled with Phrap to allow manual sequence review with Consed [49]. They were manually checked for vector at the front of the clone, the poly(A) tail was verified and removed, and the sequence was checked for any low quality bases or high quality discrepant base calls (flagged bases). Clones were rejected if they failed to contain an apparent poly(A) tail, did not have the expected vector transition at the front, or if one or more of the flagged base-pairs could not be identified with confidence.

Selected clones that did not have a good quality 3′ EST would generally not have 5′ and 3′ coverage for all bases; these sequences were validated against the B73 genome BAC sequences. A report for each clone with its alignment to a BAC was created with the phredPhrap script (available with Consed), from which navigation files were generated for Consed so that mis-matches between the clone and the BAC(s) could be reviewed as follows: the clone was determined to be correct as called, or incorrect base(s) were changed, or the clone was logged as needing an additional walk to determine the true base value. The final set of FLcDNAs was submitted with relaxed completion rules that required a Phrap calculated quality value of ≥30 for every base, ORFs with ≥100 amino acid residues, and 5′ and 3′ UTRs with ≥50 bases.

Of the walked clones, 7,903 were extended but did not reach completion status. There is valuable information in these potentially unique genes, so we offer them as part of the query-based maize FLcDNA website. Failure to complete the cDNA occurred for the following reasons: the clone contained an unverifiable section (e.g. long homopolymer or pattern repeating stretch), the clone abruptly ended without a perceptible poly(A) tail, or the clone was selected near the end of the project and remained unfinished.

### Analysis

Contaminated FLcDNAs were found by comparing them against the maize, rice and Arabidopsis rRNA sequences with a BLAST e-value≤1e-50, which identified 26 rRNAs. An additional 110 FLcDNAs were identified that encoded proteins highly similar to bacteria (16 cDNAs), fungus (76 cDNAs) and vertebrate (18 cDNAs) and did not show similarity with plant proteins.

The ORFs were computed using the software GETORF in EMBOSS package [50] with parameters “–minsize 150, -find 1, -methionine, -noreverse”. TE and SSR analyses were performed using RepeatMasker (repeatmasker.org). For TE analysis, the Poaceae (grass family) TE database was downloaded from Genetic Information Research Institute (www.girinst.org) and the FLcDNAs that had masked sequence length of ≥100 bp were used for the TE insertion analysis. SSRs with length ≥20 bp and divergence ≤10% were selected for SSR location analysis. Putative transcription factors were analyzed using BLASTx with e-value≤1e-10 against rice and Arabidopsis transcription factor proteins downloaded from PlantTFDB (planttfdb.cbi.pku.edu.cn). Any maize cDNAs showing positive matches in both rice and Arabidopsis were assigned to TF families using the PlantTFDB nomenclature.

Plant homolog analysis was conducted using BLASTx (e-value≤1e-10) to compare rice, sorghum, Arabidopsis and poplar protein sequences downloaded from the following sites: 67,393 rice (MSU release 6.0; rice.plantbiology.msu.edu), 35,899 sorghum (www.phytozome.net/sorghum), 32,615 Arabidopsis (TAIR v8.0; www.arabidopsis.org) and 45,555 poplar (genome.jgi-psf.org). The maize FLcDNAs that did not have a homolog were compared with the plant UniProt database [29], where another 147 rice, sorghum, Arabidopsis or poplar homologs were identified and removed. Then the 1,475 putative unique maize FLcDNAs were mapped to GO annotated maize gene models with ≥95% ID and ≥90% alignment length using BLAT. GO over- and under- representation analysis were performed using Cytoscape [51] with BiNGO (Biological Networks Gene Ontology, [25]) plug-in and activating a hypergeometric distribution statistical test (p-value ≤0.05) with Benjamini and Hochberg false discovery rate (FDR) correction [52] relative to GO annotated maize gene models.

For annotation of all EST and FLcDNA assemblies, the unitrans were searched against the UniProt plants database (2009-06-17) using BLASTx with e-value≤1e-20. The GO [24] annotations were extracted from the UniProt file and gene association file (ftp.ebi.ac.uk/pub/databases/GO/goa/UNIPROT), which were mapped to plant GO Slim [32]. Some of the results were computed by custom Perl scripts, and the rest were obtained from the website, as follows: Table 6 was copied from the “Advanced Summary/Example Queries” page. The number of UniProt matches for the 27k were from the “UniTrans Search”, where “Non-maize UniProt Match” was set to ‘yes’; for the non-putative, the “Match Description” was set to “not putative”. Table 8, Table 9, and the top of Table 10 can all be verified from the PAVE query system.

### FLcDNA assemblies

PAVE was used for assembling the FLcDNAs. The MegaBLAST output was filtered to remove all sequence pairs that had longer flanking regions on both sides of the overlap. The first two assemblies also rejected mixed orientation. The strict assembly filtered the MegaBLAST output for 100% identity over a minimum 50 bases and the possibility of ignoring up to 10 discrepant bases on each end. Additionally, for the 69k, it rejected matches if one clone was half the size of the other. The CAP parameters were (-p 99 -y 15 –f 2), where the –p is identity, -f is the gap size (-f 0 or 1 causes CAP3 errors), and –y is the number of bases ignored on the ends. The semi-strict assembly allowed 90% identity over a minimum 50 bases and up to 15 ignored end bases, and the CAP parameters were (-p 90 –y 15 –f 500), where the –f allowed for large gaps. The loose assembly allowed 80% identity over a minimum 50 bases and up to 350 ignored end bases (to allow for highly diverged UTRs, and the CAP parameters were (-p 80 –y 350 –f 500). PAVE uses an iterative assembly algorithm, so the 350 cutoff was only used on the last of three iterations.

The 27k assemblies used the FLcDNAs submitted to GenBank from this project, where the poly(A) tail had been removed. The clones from library B were prefixed with B (including the clones from the initial sampling) and the clones from library C were prefixed with C. The 69k assemblies used the four FLcDNA datasets from GenBank with the keyword “FLI-CDNA”, with the clones prefixed as follows: F – from the 36,565 set [14], M – from the 2,073 set [13], W – from the 3,384 set [12], and BC – from both the B and C libraries of the 27,455 set. The clones have all been liberally trimmed to remove apparent poly(A) tail and slippage; trimmed clones are suffixed with ‘t’. A few clones have been reversed if the poly(A) tail seemed to be on the 5′ end; these clones are suffixed with ‘r’.

## Supporting Information

Table S1 Putative transcription factors (TF) with maize FLcDNAs.
(0.08 MB DOC)

Table S2 Summary of single-locus FLcDNAs and density by chromosomes.
(0.06 MB DOC)

Table S3 Number of gaps in FLcDNA contigs.
(0.06 MB DOC)
