# Supplementary material for: Sequencing, Mapping, and Analysis of 27,455 Maize Full-Length cDNAs
Source: PLoS Genet. 2009 Nov 20;5(11):e1000740. doi: 10.1371/journal.pgen.1000740 (PMC2774520; doi:10.1371/journal.pgen.1000740)
Supplement: Table S1 — Putative transcription factors (TF) with maize FLcDNAs. (0.08 MB DOC) [file pgen.1000740.s001.doc]

Table S1. Putative transcription factors (TF) with maize FLcDNAs.

| **TF family** | **#cDNAs** | **%** | **TF family** | **#cDNAs** | **%** |
| --- | --- | --- | --- | --- | --- |
| bHLH | 141 | 7.2 | CCAAT-HAP2 | 20 | 1.0 |
| MYB | 137 | 7.0 | HMG | 20 | 1.0 |
| bZIP | 127 | 6.5 | ZF-HD | 20 | 1.0 |
| HB | 103 | 5.2 | ZIM | 18 | 0.9 |
| C3H | 96 | 4.9 | AS2 | 16 | 0.8 |
| AP2/EREBP | 90 | 4.6 | CPP | 15 | 0.8 |
| NAC | 85 | 4.3 | C2C2-YABBY | 15 | 0.8 |
| WRKY | 82 | 4.2 | BES1 | 15 | 0.8 |
| C2H2 | 64 | 3.3 | JUMONJI | 14 | 0.7 |
| MADS | 61 | 3.1 | LIM | 14 | 0.7 |
| GARP-G2-like | 53 | 2.7 | ARID | 10 | 0.5 |
| AUX/IAA | 49 | 2.5 | EIL | 9 | 0.5 |
| GRAS | 47 | 2.4 | CCAAT-HAP5 | 8 | 0.4 |
| MYB-related | 45 | 2.3 | GeBP | 8 | 0.4 |
| PHD | 44 | 2.2 | GARP-ARR-B | 8 | 0.4 |
| C2C2-CO-like | 44 | 2.2 | E2F/DP | 8 | 0.4 |
| FHA | 41 | 2.1 | BBR/BPC | 7 | 0.4 |
| LUG | 38 | 1.9 | PLATZ | 6 | 0.3 |
| SBP | 35 | 1.8 | Nin-like | 6 | 0.3 |
| ARF | 34 | 1.7 | CCAAT-HAP3 | 5 | 0.3 |
| GRF | 34 | 1.7 | TAZ | 5 | 0.3 |
| PcG | 33 | 1.7 | GIF | 5 | 0.3 |
| TCP | 29 | 1.5 | VOZ | 5 | 0.3 |
| ABI3/VP1 | 26 | 1.3 | CAMTA | 4 | 0.2 |
| C2C2-Dof | 26 | 1.3 | SRS | 4 | 0.2 |
| Trihelix | 26 | 1.3 | MBF1 | 4 | 0.2 |
| TLP/TUB | 26 | 1.3 | PBF-2-like-Whirly | 4 | 0.2 |
| HSF | 23 | 1.2 | CCAAT-Dr1 | 4 | 0.2 |
| C2C2-GATA | 23 | 1.2 | ULT | 3 | 0.2 |
| Alfin | 22 | 1.1 | HRT-like | 1 | 0.1 |
|  |  |  |  | 1965 | 100.0 |
