# Supplementary material for: Sequencing, Mapping, and Analysis of 27,455 Maize Full-Length cDNAs
Source: PLoS Genet. 2009 Nov 20;5(11):e1000740. doi: 10.1371/journal.pgen.1000740 (PMC2774520; doi:10.1371/journal.pgen.1000740)
Supplement: Table S2 — Summary of single-locus FLcDNAs and density by chromosomes. (0.06 MB DOC) [file pgen.1000740.s002.doc]

Table S2. Summary of single locus FLcDNAs and density by chromosomes.

| **Chromosome** | **Length (Mb)** | **# of cDNA** | **#cDNA/Mb** | **kb/cDNA** |
| --- | --- | --- | --- | --- |
| 1 | 300.2 | 3880 | 12.9 | 77.4 |
| 2 | 234.8 | 2800 | 11.9 | 83.8 |
| 3 | 230.6 | 2686 | 11.6 | 85.8 |
| 4 | 247.1 | 2482 | 10.0 | 99.6 |
| 5 | 216.9 | 2923 | 13.5 | 74.2 |
| 6 | 169.3 | 2092 | 12.4 | 80.9 |
| 7 | 171.0 | 2007 | 11.7 | 85.2 |
| 8 | 174.5 | 2212 | 12.7 | 78.9 |
| 9 | 152.4 | 1691 | 11.1 | 90.1 |
| 10 | 149.7 | 1581 | 10.6 | 94.7 |
| Total | 2046.3 | 24354 | 11.9 | 84.0 |
