# Supplementary material for: Sequencing, Mapping, and Analysis of 27,455 Maize Full-Length cDNAs
Source: PLoS Genet. 2009 Nov 20;5(11):e1000740. doi: 10.1371/journal.pgen.1000740 (PMC2774520; doi:10.1371/journal.pgen.1000740)
Supplement: Table S3 — Number of gaps in FLcDNA contigs. (0.06 MB DOC) [file pgen.1000740.s003.doc]

Table S3. Number of gaps in FLcDNA contigs.

| **Num**  **Gap** | **27k assembly** | | **69k assembly** | |
| --- | --- | --- | --- | --- |
|  | **semi** | **loose** | **semi** | **loose** |
| 1 | 493 | 915 | 3098 | 3337 |
| 2 | 84 | 347 | 1611 | 2061 |
| 3 | 32 | 234 | 923 | 1479 |
| 4 | 26 | 140 | 572 | 991 |
| 5 | 19 | 138 | 385 | 746 |
| 6 | 10 | 134 | 234 | 525 |
| 7 | 4 | 87 | 160 | 405 |
| 8 | 6 | 80 | 109 | 322 |
| 9 | 3 | 64 | 72 | 229 |
| 10 | 1 | 50 | 37 | 175 |
| 11 | 1 | 30 | 25 | 137 |
| 12 | 0 | 30 | 16 | 120 |
| 13 | 1 | 16 | 9 | 76 |
| 14 | 1 | 14 | 8 | 57 |
| 15 | 0 | 12 | 5 | 50 |
| 16 | 1 | 9 | 2 | 34 |
| 17 | 1 | 5 | 4 | 27 |
| 18 | 0 | 3 | 6 | 24 |
| 19 | 0 | 5 | 1 | 14 |
| 20 | 0 | 4 | 0 | 15 |
| 21 | 0 | 3 | 0 | 13 |
| 22 | 1 | 2 | 0 | 9 |
| 23 | 0 | 2 | 0 | 5 |
| 24 | 0 | 3 | 1 | 6 |
| 25 | 0 | 0 | 0 | 10 |
